# Modelling hypersensitivity to trastuzumab defines biomarkers of response in HER2 positive breast cancer

**DOI:** 10.1186/s13046-021-02098-z

**Published:** 2021-10-07

**Authors:** Laura Díaz-Gil, Fara Brasó-Maristany, Claudriana Locatelli, Ariana Centa, Balász Győrffy, Alberto Ocaña, Aleix Prat, Atanasio Pandiella

**Affiliations:** 1 Instituto de Biología Molecular y Celular del Cáncer, CSIC and CIBERONC, Institute of Biomedical Research of Salamanca (IBSAL), Salamanca, Spain; 2 Translational Genomics and Targeted Therapies in Solid Tumors, August Pi i Sunyer Biomedical Research Institute (IDIBAPS), Barcelona, Spain; 3 Department of Medical Oncology, Hospital Clínic of Barcelona, Barcelona, Spain; 4 Programa de Pós-Graduação em Desenvolvimento e Sociedade, Universidade Alto Vale do Rio do Peixe – UNIARP, Caçador, SC Brazil; 5 Department of Bioinformatics and 2nd Department of Pediatrics, Semmelweis University and TTK Cancer Biomarker Research Group, Budapest, Hungary; 6 San Carlos University Hospital, Madrid, Spain; 7 SOLTI cooperative group, Barcelona, Spain; 8 Department of Oncology, Quironsalud Group, IOB Institute of Oncology, Barcelona, Spain; 9 Department of Medicine, University of Barcelona, Barcelona, Spain

**Keywords:** Breast cancer, Trastuzumab, Hypersensitive, Response, Biomarker

## Abstract

**Background:**

Trastuzumab-based therapies are the therapeutic option for HER2 positive (HER2+) breast cancer. HER2 amplification is the only biomarker validated for trastuzumab-based therapies. However, a proportion of tumors become refractory during treatment course. For this reason, the finding of new biomarkers beyond HER2 overexpression to identify patients who would benefit most from trastuzumab regimens is of outstanding importance.

**Methods:**

Models of trastuzumab-resistant or hypersensitive cells were generated by exposure to trastuzumab. Cell surface, total HER2, and analyses of proteins involved in cell cycle or apoptosis were analyzed by western blotting. Cell proliferation was analyzed by cell counting, cell cycle and apoptosis was evaluated by FACS. Transcriptomic characterization of the cellular models was performed using bioinformatic online tools, and clinico-genomic analyses were performed using the PAMELA clinical trial data.

**Results:**

Besides differing in sensitivities to trastuzumab, the different cellular models also showed distinct response to other anti-HER2 drugs (lapatinib, neratinib, pertuzumab and T-DM1) used in the clinic. That differential effect was not due to changes in cell surface, total or activated HER2. Trastuzumab caused important induction of cell death in hypersensitive cells but not in parental or resistant cells. Transcriptomic analyses of these cellular models together with querying of online databases allowed the identification of individual genes and gene signatures that predicted prognosis and trastuzumab response in HER2+ breast cancer patients.

**Conclusion:**

The identification of trastuzumab response biomarkers may be used to select patients particularly sensitive to facilitate the use of trastuzumab-based therapies and refine follow-up guidelines in patients with HER2+ tumors.

**Supplementary Information:**

The online version contains supplementary material available at 10.1186/s13046-021-02098-z.

## Background

The finding that the transmembrane tyrosine kinase HER2 is overexpressed in 15–25% of human breast tumors together with correlation with aggressive tumor growth and poor prognosis [1] fostered the development of agents to target that protein with therapeutic purposes [2]. Two types of agents that target HER2 have reached the oncology clinic [3] On one hand, antibodies that recognize the extracellular region of HER2. The first such agent was trastuzumab, a humanized monoclonal antibody against HER2 [4, 5]. Several seminal clinical studies demonstrated its efficacy, improving overall survival of HER2-positive breast cancer patients [6–9]. Based on these early reports, the actual standard treatment of HER2 positive early breast cancer includes trastuzumab combined with taxanes or dual HER2 blockade, using trastuzumab and chemotherapy in combination with pertuzumab, another HER2-directed antibody [10]. Metastatic patients also receive dual HER2 blockade plus chemotherapy as first-line treatment [11, 12]. Derivatives of trastuzumab such as trastuzumab-emtansine (T-DM1) or trastuzumab-deruxtecan, two drugs that belong to the antibody-drug conjugates class of antitumorals, have also been approved as a second or third-line treatment for metastatic HER2 positive breast cancer patients [13–15]. Moreover, bispecific antibodies directed to two different targets or epitopes are being developed as a novel strategy to overcome trastuzumab resistance [16, 17]. The bispecific antibody M802 recognized HER2 as trastuzumab and recruits CD3-positive immune cells, increasing cytotoxicity, eliminating HER2+ tumor cells [18].

The second group of anti-HER2 agents includes membrane-permeant, small molecule tyrosine kinase inhibitors (TKIs) such as lapatinib, neratinib and tucatinib. Treatment with neratinib after adjuvant trastuzumab-based therapy demonstrated clinical benefit [19]. Additional treatment options for patients who become refractory to the standard therapies include lapatinib plus capecitabine, combinations of trastuzumab with other chemotherapies (vinorelbine or gemcitabine), or combinations of trastuzumab and lapatinib without chemotherapy [8, 20, 21]. Recently, tucatinib has been approved for use in combination with trastuzumab and capecitabine for advanced unresectable or metastatic tumors in third-line setting [22].

The main criteria for selection of patients to be treated with drugs that target HER2 is HER2 overexpression, which is routinely detected by immunohistochemistry or in situ hybridization [23]. However, while most patients respond to trastuzumab-based therapies, some patients exhibit intrinsic or acquired resistance to this drug and the disease relapses, especially in the advanced stages. Therefore, there must be additional factors, besides HER2 overexpression, that dictate sensitivity to trastuzumab-based therapies. Considering that, we initiated a study aimed at discovering additional biomarkers beyond HER2 amplification that can identify patients who are likely to benefit most from trastuzumab-based treatments and predict pathological complete response. To that end, we used several cellular models derived from the HER2 positive breast cancer cell line BT474. This cell line has been widely used as a model for the analysis of the pathophysiological role of HER2 in breast cancer, including studies on the antitumoral action of drugs that target HER2, such as trastuzumab [24–26]. Using that cell lines as a backbone, we isolated resistant and hypersensitive derivatives, that were characterized biologically and genomically. The use of these models, together with querying of public databases containing clinic-genomic data, led to the identification of several genes linked to both prognosis and trastuzumab response. Moreover, we report the identification of gene sets enriched in patients who benefited most from a dual HER2 blockade.

## Methods

### Reagents and antibodies

Dulbecco’s modified Eagles Medium (DMEM), fetal bovine serum (FBS), penicillin and streptomycin and trypsin-EDTA were purchased from Life Technologies (Carlsbad, USA). Protein A-Sepharose was from GE Healthcare Life Sciences (Piscataway, USA) and 4′,6-diamidino-2-phenylindole (DAPI) were purchased from Sigma-Aldrich (St Louis, USA). Other generic chemicals were purchased from Sigma, Roche Biochemicals (St Louis, USA) or Merck (Darmstadt, Germany). Lapatinib and neratinib were from LC Laboratories (Woburn, USA) and trastuzumab, pertuzumab and T-DM1 was purchased from a local pharmacy.

The anti-pTyr (sc-7020), anti-MCL1 (sc-819) and anti-PARP (sc-8007) antibodies were purchased from Santa Cruz Biotechnology (Santa Cruz, USA); anti-BCLX (610211), anti-caspase 3 (610323) and anti-BAX (556467) from BD Biosciences (Palo Alto, USA); anti-p27 (#3686), anti-cleaved Caspase 3 (#9664), anti-Caspase 8 (#9746), anti-Caspase 9 (#9502), anti-Caspase 7 (#9492), anti-BID (#2002), anti-p21 (#2947) and anti-HER2 (#2165) for immunofluorescence from Cell Signaling Technologies (Beverly, USA); anti-HER2 (OP15) from Calbiochem (La Jolla, USA) and anti-Calnexin (SPA-860) from Stressgen Bioreagents Corporation (British Columbia, Canada); The horseradish peroxidase conjugates of anti-rabbit (170–6515) or anti-mouse (NA931) immunoglobulin G were from Bio-Rad Laboratories (Hercules, USA) or from GE Healthcare Life Sciences (Piscataway, USA), respectively; anti-Rabbit-Cy3 (611–165-215), anti-human-Alexa488 (609–545-213) and anti-human-Cy3 (109–165-003) from Jackson Immunoresearch (West Grove, USA).

### Cell culture and generation of trastuzumab-resistant and trastuzumab-hypersensitive models

BT474 and SKBR3 cells were obtained from the ATCC. BTRH cells were generated as described [27]. With the aim to generate trastuzumab hypersensitive cells, BT474 cells were seeded at low density and two different strategies were followed: 1) Clones were picked and trastuzumab response was analyzed. A highly sensitive clone termed BTSH was isolated; 2) Cells were chronically treated with trastuzumab 50 nM for 2 months. Fifty-four clones were isolated and trastuzumab response was assessed. One (ST35) out of 54 was hypersensitive to trastuzumab. All cell lines were grown in DMEM medium supplemented with 10% FBS and antibiotics (penicillin 10000 U/ml, streptomycin 100 μg/ml). Cells were cultured at 37 °C in a humidified atmosphere in the presence of 5% CO_2_ and 95% air.

### Immunofluorescence and phase contrast microscopy

The immunofluorescence protocol was performed as previously described [28]. Images were acquired by confocal IF microscopy using a Leica TSC SP5 system (Leica Microsystems CMS, Wetzlar, Germany).

### Measurement of HER2 cell surface levels

To measure HER2 surface levels, cells were processed as detailed in [29]. In brief, detached cells were incubated with trastuzumab 10 nM for 30 min and then with anti-human Cy3-conjugated secondary antibody (1:80 dilution). Labelled cells were analyzed by flow cytometry using BD Accuri™ C6 cytometer (BD Biosciences, Palo Alto, USA).

### Cell proliferation, BrdU incorporation, cell cycle and apoptosis

Cell proliferation was determined by cell counting experiments [30]. Cells were seeded in 6-well plates at a density of 40,000 or 200,000 cells/well in the case of BT474; 30,000 or 150,000 BTRH cells/well; 60,000 or 200,000 BTSH cells/well. Lower cell density was used for long-time experiments. The following day, medium was replaced with fresh DMEM containing the indicated drug: trastuzumab, pertuzumab, T-DM1, lapatinib or neratinib, at concentrations indicated in the figures. Cells were cultured for 7 days in the case of trastuzumab and pertuzumab or 3 days for the other drugs. To perform cell counting, cells were detached with trypsin-EDTA and diluted in ISOTON® (Beckman Coulter Life Sciences). The number of cells per well was measured using a Z1 Coulter Particle Counter (Beckman Coulter).

The number of non-viable cells after 6 days trastuzumab 50 nM treatment was determined following the protocol of the FITC Annexin V Apoptosis Detection Kit I (BD Biosciences) as previously described [31]. For cell cycle analysis, cells were incubated with trastuzumab 50 nM for 6 days and stained with propidium iodide as described [32]. Caspase 3 activity after 6 days trastuzumab 50 nM treatment was fluorometrically determined by the caspase 3 activity reporter Ac-DEVD-AFC (BD Biosciences, Palo Alto, USA) as described [29]. Percentage of cells entry in S phase after 120 h trastuzumab 50 nM incubation was determined by flow cytometry following the protocol of the FITC-BrdU Flow Kit (BD Biosciences) using a BD Accuri™ C6 cytometer. BT474, BTRH and BTSH cells were seeded in 60 mm plates at a density of 100,000 and received 10 μM BrdU pulse for 12 h.

### Immunoprecipitation and Western blotting

Protein extraction, immunoprecipitation and Western blot protocols have been described [33]. In some cases, proteins were transferred from gels using the Trans Blot Turbo Mini 0.2 μm PVDF Transfer Packs kits in a TransBlot® Turbo™ Transfer System from Bio-Rad Laboratories (Hercules, USA).

### Microarrays analysis of mRNA

RNA was isolated using TRIzol™ reagent (Invitrogen, Carlsbad) according to manufacturer’s instructions. Next, RNA purification, cDNA synthesis and integrity were assessed as described in [34]. cDNA was hybridized to HuGene 2.0 ST microarrays (Affymetrix, Santa Clara, USA). Raw. CEL files were normalized using Expression Console 1.4 software (Affymetrix). Then, the differential gene expression analysis was performed using the Transcription Analysis Console 4.0 software (Affymetrix). Cut-off for differentially expressed genes was fold change ≥2 and *p*-value ≤0.05.

Microarray data from BT474 and BTRH cells were previously available in GEO database (GSE119397 [27],).

### qRT-PCR

RNA was isolated using the PureLink RNA Mini Kit from Life technologies (Carlsbad, USA) following the manufacturer’s instructions. First-strand cDNA was synthesized by M-MLV reverse transcriptase (Invitrogen, Carlsbad). Real time PCR reactions were performed on an iQ5 Real Time PCR Detection System using SYBR Green Master Mix, both from Bio-Rad Laboratories. Gene expression was normalized to the housekeeping GAPDH and relativized to BT474. mRNA levels are shown as fold change relativized to the expression of BT474. The primers used for gene amplification were:

- PTPRM (FW: 5′-GGCAAGTCAAGTCCAAGAGC-3′; RV: 5′-CTGTTTGCACCATGTTCACC-3′)
- STC1 (FW: 5′- CTCAGGGAAAAGCATTCGTC -3′; RV: 5′-AGCTGGACGACCTCAGTGAT-3′)
- SCGB2A2 (FW: 5′-GCTGCCCCTTATTGGAGAAT-3′; RV: 5’TGCTCAGAGTTTCATCCGTTT-3′)
- BRINP2 (FW: 5′-GGCTGCAGCAACTATGACAA-3′; RV: 5′-TTGCACCAGCAGTCATTCTC-3′)
- GRIK3 (FW: 5′-CACCACAGATGACCGTGAAC-3′; RV: 5′-TTTCAGGCTGATGATGTCCA-3′)
- DTNA (FW: 5′-GTTCCCAGATCAGCCTGAGA-3′; RV: 5′-GGACTTCCTGAGGAGGGAAC-3′)
- LRP1B (FW: 5′-TGGATATGCGATGGTCAGAA-3′; RV: 5′-TATGGCACCACAAATGCTGT-3′)
- PDE7B (FW: 5′-TTGCCAAAGGAAATGACACA-3′; RV: 5′-CACACTTCAAGGCGATCTGA-3′)
- IL20RA (FW: 5′-CTGTTGTCCTGACAGCTCCA-3′; RV: 5′-AGTCCTGGCACACTGCTTCT-3′)
- FBXL7 (FW: 5′-TGTGTCTCATGCTGGAAACC-3′; RV: 5′-CAGATTAGGGCAGAGGGACA-3′)
- TXNIP (FW: 5′-GCCACACTTACCTTGCCAAT-3′; RV: 5′-GTTGCAGCCCAGGATAGAAG-3′)
- ATXN3 (FW: 5′-ATTGCGAAGCTGACCAACTC-3′; RV: 5′-ATTCCTGAGCCATCATTTGC-3′)
- CXCL17 (FW: 5′-GCCAAGAATGTGAGTGCAAA-3′; RV: 5′-TGCTTGTTTGGCTTTCTGTG-3′)
- IFI6 (FW: 5′-GGTGGAGGCAGGTAAGAAAA-3′; RV: 5′-ATCGCAGACCAGCTCATCA-3′)
- NPNT (FW: 5′-TACACCAAAGCCAACACCAA-3′; RV: 5′-TGGGTTTCTGAGGGTCTGTC-3′)
- ELMO1 (FW: 5′-GGAGAAACGCAAGTCCATGT-3′; RV: 5′-CAATCCGGATGTAGGCATCT-3′)
- GRIK2 (FW: 5′-TCAGACGTGGTGGAAAACAA-3′; RV: 5′-ACTGTCAGAAAGGCGGCTAA-3′)
- UPK1A (FW: 5′-TGGTAGCCAGTTTTGGTGTG-3′; RV: 5′-AAGGTCAGCATCTGCTTGGT-3′)
- PPP1R3C (FW: 5′-ACCATGCTAATGGGCAAGTC-3′; RV: 5′-AGGAGACGTCTGGTGGAATG-3′)
- HMGCS2 (FW: 5′-CCAGCAGTGACACACAAACC-3′; RV: 5′-CCCATTGTGAGTGGAGAGGT-3′)
- CEACAM7 (FW: 5′-GAACCCTGCTGATCCAGAAC-3′; RV: 5′-CTCCACCGGATTGAAGTTGT-3′)
- PIP (FW: 5′-CGTCCAAATGACGAAGTCAC-3′; RV: 5′-GGGGATTACAGCAGCATCAT-3′)
- LUZP2 (FW: 5′-TTGAGAGTCACAGGCAGTGG-3′; RV: 5′-TTGAGAGTCACAGGCAGTGG-3′)
- IL1R1 (FW: 5′-TGTGATTGTGAGCCCAGCTA-3′; RV: 5′-TGTTTGCAGGATTTTCCACA-3′)
- CEACAM5 (FW: 5′-TATTACCGTCCAGGGGTGAA-3′; RV: 5′-TTGGCCTGGCAGGTATAGAG-3′)
- ABCA5 (FW: 5′-GGACCCCTGTTCTCGACATA-3′; RV: 5′-CCGATCCCCCATTTACTTTT-3′)
- SCGN (FW: 5′-TTTGCACAAGGTGAAACAGC-3′; RV: 5′-GCGCCAAATCTGCATAAACT-3′)
- SPINK8 (FW: 5′-GACGCCATCCTTGTTCTAGC-3′; RV: 5′-TAACCTGGTCACTGCCACAA-3′)
- CYSLTR1 (FW: 5′-TGACCGCTGCCTTTTTAGTC-3′; RV: 5′-ATGCAGCCAGAGACAAGGTT-3′)
- GAPDH (FW: 5′-GAGTCAACGGATTTGGTCGT-3′; RV: 5′-GATCTCGCTCCTGGAAGATG-3′)

### Outcome analyses

The Kaplan Meier Plotter (KM plotter) online tool [35] was used to determine the prognostic power of genes in a cohort of HER2+ breast cancer patients. Patients were splitted in low or high expression by trichotomization by lower quartile versus upper quartile (Q1 vs Q4) and analysis was restricted to HER2+ subtype. Survival was assessed by RFS. Probes used for each gene were 230109_at (*PDE7B*, 358 patients), 204513_s_at (*ELMO1*, 695 patients), 213845_at (*GRIK2*, 695 patients), 214624_at (*UPK1A*, 695 patients). For multiple gene analysis, same cut-off and probes were used. KM plotter calculated the mean expression of selected probes (358 patients). ROC plotter database [36] was used to link gene expression and trastuzumab response in a cohort of HER2+ breast cancer patients. Sensitivity, specificity and area under the curve (AUC) were computed to evaluate the efficiency of the biomarker candidates. AUC value was computed instead of accuracy as AUC values are independent of the used cut-off. Significant genes were selected if their high or low levels and prognosis correlation were consistent to their expression values in BTRH and BTSH cell lines, meaning better outcome if upregulated in BTSH or the opposite.

### Predictive analyses with PAMELA patients

Association between differentially expressed genes in BTRH or BTSH versus the parental BT474 cell line and trastuzumab response was evaluated using genomic data from HER2+ breast cancer patients tumors from the PAMELA clinical trial (*n* = 151, including patients positive for hormone receptor). The tumors were previously analyzed using the nCounter platform (NanoString Technologies, Seattle, Washington, USA) that included a panel of 560 genes [37, 38]. The overlapping of DEGs identified by microarrays analyses and genes analyzed in tumoral samples led to the generation to a trastuzumab-hypersensitive and a trastuzumab-resistant gene signatures (Fig. 5A).

The mean expression of the upregulated genes and downregulated genes in BTRH and BTSH was explored in the baseline samples of the PAMELA trial and associated with variables of response to anti-HER2 therapy (pathological complete response (pCR) at surgery, residual cancer burden (RCB) at surgery, and the normal-like subtype at day 14 of treatment). The PAM50 signature was determined as previously described [39].

### Statistical analyses

Comparisons of continuous variables between two groups were performed using a two-sided Student’s *t* test. Differences were considered statistically significant when *p*-values were less than 0.05. Statistical data are presented as the mean ± SD. All data were analyzed using the software GraphPad Prism 8 (San Diego, USA). Comparisons of signature BTSH or BTRH scores across clinical and biological values of PAMELA’s patients samples were determined using two-tailed *t*-tests and one-way ANOVA. All differences were considered significant at *p*-value < 0.05. These statistical computations were carried out in R 4.0.3 (http://cran.r-project.org).

## Results

### Generation of trastuzumab-resistant and trastuzumab-hypersensitive HER2+ breast cancer cells

**Fig. 1:**
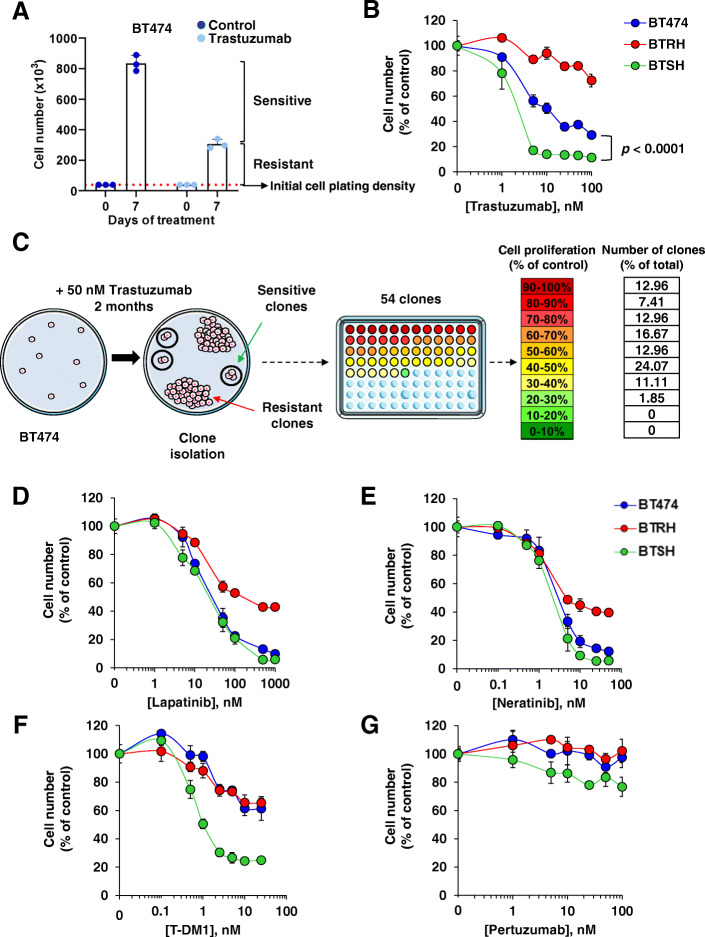
Generation and characterization of trastuzumab-resistant and hypersensitive cells. **A** Proliferation of BT474 cells in the presence of trastuzumab. Cells were treated with trastuzumab 50 nM for 7 days and cell number was measured by cell counting experiments. Treatment affects a population of sensitive cells while a resistant population escaped form trastuzumab action. Data are represented as mean ± SD, normalized to untreated controls. **B** Response of BT474 and their trastuzumab-resistant and hypersensitive models to trastuzumab assessed in dose-response experiments by cell counting at 7 days. Data are represented as mean ± SD, normalized to untreated controls. **C** Schematic representation of clones with distinct trastuzumab sensitivity generation. BT474 cells were seeded at low density and treated with trastuzumab for 2 months. Fifty-four clones were isolated and cell proliferation in the presence of trastuzumab was evaluated. Colors in the 96 well plate represents the proliferation rate after trastuzumab treatment of each clone as indicated. The percentage of clones belonged to each proliferation group normalized to the total amount of clones is indicated. Susceptibility of BT474, BTRH and BTSH to lapatinib (**D**), neratinib (**E**) and T-DM1 (**F**) treatment for 3 days and pertuzumab (**G**) for 7 days was determined by dose-response assays. Cell number was determined by cell counting assay. Data are represented as mean ± SD of triplicates, normalized to untreated controls

The effect of trastuzumab on cell proliferation was initially analyzed in BT474 and SKBR3 HER2+ breast cancer cell lines, two cellular models which have widely been used to study HER2 biology and pharmacology. In cell proliferation experiments, a seven-day treatment of wild type BT474 cells with trastuzumab resulted in a decrease in the number of cells when compared to the number of cells present in untreated BT474 cultures (Fig. 1A). Trastuzumab, while reducing the number of cells, did not fully provoke disappearance of the whole BT474 cell population, even after prolonged treatment with the drug. Moreover, those experiments showed that at the end of a seven-day experiment, the number of BT474 cells in the cultures treated with trastuzumab was higher than the number of cells present at the start of the experiment, suggesting that at least some cells proliferate even in the presence of the drug. Similar responses were found in SKBR3 cells (Figure S1A). Together, the above data suggested the presence of a population of sensitive cells which was affected by trastuzumab, as well as a population of cells which were less affected or even resistant to the drug (Fig. 1A and Figure S1A).

The strategy to identify genes that could be used to predict trastuzumab response in HER2+ breast cancer initiated by creating models of sensitivity and resistance to this drug. To that end we used two parallel approaches. In one of them, clones of BT474 cells were directly picked and tested for their sensitivity to trastuzumab. These studies succeeded in the isolation of a clone, hereon termed BTSH (from BT474 cells Sensitive to Herceptin™) that showed increased response to trastuzumab when compared to the original BT474 cells (Fig. 1B). In these experiments we also used BTRH cells as a model for trastuzumab resistance, whose generation has previously been described [27]. The latter showed substantially less sensitivity to trastuzumab than the parental cells.

In a second experimental setting, BT474 cells were plated at low density and treated with trastuzumab (50 nM) for 2 months (Fig. 1C). As a control, untreated BT474 cells were used. Two types of responses were expected. One representing trastuzumab-resistant cells, characterized by clones which grew at similar rates as cells growing in untreated plates. The second expected condition was that of clones sensitive to trastuzumab which would duplicate much less and create colonies with a small number of cells. Fifty-four clones were isolated and their response to a saturating dose of trastuzumab (50 nM) analyzed (Fig. 1C and Figure S1B and C). Eighteen of the isolated clones resulted more resistant than parental BT474 cells (Fig. 1C and Figure S1B and C). Twenty clones responded to trastuzumab similarly to wild type cells, and one clone (ST35) had a higher sensitivity to trastuzumab when compared to parental BT474 cells (Figure S1B-D). These results demonstrated that the heterogeneous nature of the response to trastuzumab in cultured BT474 cells was due to the presence of clones with different sensitivity to trastuzumab. Moreover, these experiments allowed the isolation of clones of BT474 cells hypersensitive to trastuzumab.

### Response of trastuzumab-resistant and hypersensitive cells to other agents that target HER2

The response of cells isolated for their resistance or hypersensitivity to trastuzumab to other agents used in the breast cancer clinic was then evaluated, as they are indicated either in combination with trastuzumab or after trastuzumab-based therapies. The TKIs, lapatinib and neratinib exerted a similar effect on BTSH and BT474 cells (Fig. 1D and E). However, BTRH cells were more resistant to the action of these drugs, as previously reported [29] The antibody drug conjugate T-DM1 had a similar effect on BT474 and BTRH cells but was more potent and efficient on BTSH (Fig. 1F). Pertuzumab did not affect the proliferation of any of the three cell lines (Fig. 1G). These studies led to the conclusion that the increased sensitivity of BTSH cells to trastuzumab is shared with the trastuzumab-derivative T-DM1, but not with TKIs, which acted similarly on parental and BTSH cells.

### Characterization of trastuzumab-hypersensitive cells

**Fig. 2:**
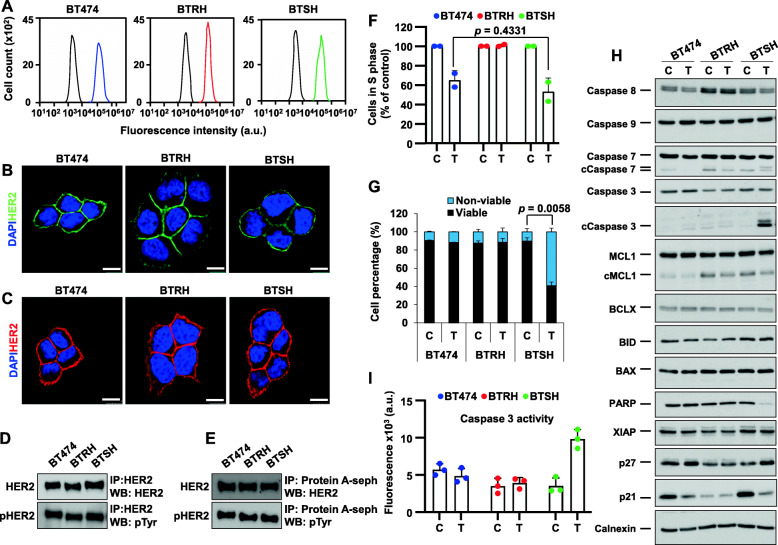
Trastuzumab causes cell cycle arrest and cell death in trastuzumab-hypersensitive cells. Trastuzumab-resistant and trastuzumab-hypersensitive models maintain HER2 levels (**A**-**E**). HER2 surface levels were analyzed by flow cytometry (**A**), immunofluorescence (**B**) and cell surface immunoprecipitation (**E**) using trastuzumab 10 nM as primary antibody in all cases. Total HER2 levels were determined by immunofluorescence (**C**) or western blot (**D**). Levels of total (**D**) and cell surface tyrosine-phosphorylated HER2 were determined by western blot. For immunofluorescence, cells were fixed and stained for HER2 using trastuzumab (green) (**B**) or an anti-HER2 antibody (red) (**C**) and DNA (blue). Scale bar, 10 μm. **F** Evaluation of S phase entry upon trastuzumab treatment. Cells were treated with trastuzumab for 5 days, received a 10 μM pulse of BrdU and BrdU incorporation was determined by flow cytometry. Data is represented as the mean ± SD, normalized to untreated controls of two independent experiments. **G** Effect of trastuzumab on cell death. Cells were treated with trastuzumab for 6 days and stained with annexin V-FITC and propidium iodide, followed by flow cytometry analysis. The graph shows mean ± SD of viable and non-viable cells from two independent experiments. **I** Trastuzumab treatment induces caspase 3 activation in BTSH cells. Cells were treated with trastuzumab for 6 days and caspase 3 activity was determined by fluorometric techniques using a caspase 3 specific substrate. **H** Analysis of proapoptotic and cell cycle progression proteins after trastuzumab treatment. Cells were treated with trastuzumab for 6 days and the amount of the indicated proteins was assessed by western blot. cCaspase stands for cleavage caspase. Calnexin was used as a loading control. C: control; T: trastuzumab; IP: immunoprecipitation; WB: western blot

The mechanisms behind the differential sensitivity to trastuzumab of BT474, BTRH and BTSH cells was next explored. Since loss or truncation of HER2 has been linked to resistance to trastuzumab [40–43], total or cell surface HER2 was analyzed. Cytometric (Fig. 2A) or immunofluorescent staining (Fig. 2B) showed that the three cell lines expressed indistinguishable levels of cell surface HER2. Moreover, immunofluorescent analyses showed similar levels and distribution of total HER2 (Fig. 2C). Western blotting studies of total or tyrosine-phosphorylated HER2 confirmed that the three cell lines expressed similar levels (Fig. 2D). Additional western blotting analyses of cell surface HER2 and its tyrosine phosphorylated form also showed similar levels in the three cell lines (Fig. 2E). Therefore, these analyses indicated that the differences in the response to trastuzumab of the three cell lines were not due to changes in the amount of total, cell surface, or tyrosine phosphorylated HER2.

To analyze whether the reduction in cell number caused by trastuzumab was due to cell cycle blockade, increased cell death or a combination of both, studies on the action of the drug on these cellular properties were carried out. Regular staining with propidium iodide (PI) showed that trastuzumab increased the amount of BT474 and BTSH cells in the G1 phase (Figure S2A). In contrast, no increase in G1 was observed in BTRH cells. BrdU incorporation studies to explore entry of cells into S phase indicated that BT474 and BTSH cells had similar sensitivity to trastuzumab, which reduced BrdU incorporation (Fig. 2F). No differences in BrdU incorporation was observed in BTRH cells treated with trastuzumab, when compared to untreated controls. Western blotting analyses of p27, which has been involved in cell cycle arrest upon treatment with trastuzumab, showed that the drug induced upregulation in BT474 and BTSH cells but not in BTRH cells (Fig. 2H). Interestingly, p21 expression was downregulated by trastuzumab in both BT474 and BTSH cells. BTRH cells expressed lower resting levels of p21 and those levels were insensitive to trastuzumab.

Double Annexin-V/PI staining showed that trastuzumab treatment provoked a small increase in the amount of annexin-V positivity in BT474 cells (Figure S2B). A similar discrete increase in dead cells caused by trastuzumab was also observed in SKBR3 cells (Figure S2C). The proapoptotic effect of trastuzumab on BTSH cells was more pronounced than in BT474 cells (Fig. 2G). In contrast, no detectable effect of trastuzumab was observed on BTRH cells. Biochemically, in BTSH cells, treatment with trastuzumab increased cleavage of caspase 3, caspase 7 and PARP (Fig. 2H). Analyses of the activity of caspase 3 indicated that trastuzumab significantly increased its activity (Fig. 2I). However, this effect was not detected in BT474 or BTRH cells. Together, the above data confirmed that BTRH cells offered resistance to cell cycle blockade or apoptosis induced by trastuzumab. The results also demonstrated that the increased sensitivity of BTSH cells to trastuzumab was likely due to a higher sensitivity of those cells to the proapoptotic effect of the drug.

### Transcriptomic profile of trastuzumab-resistant versus trastuzumab-hypersensitive cells

**Fig. 3:**
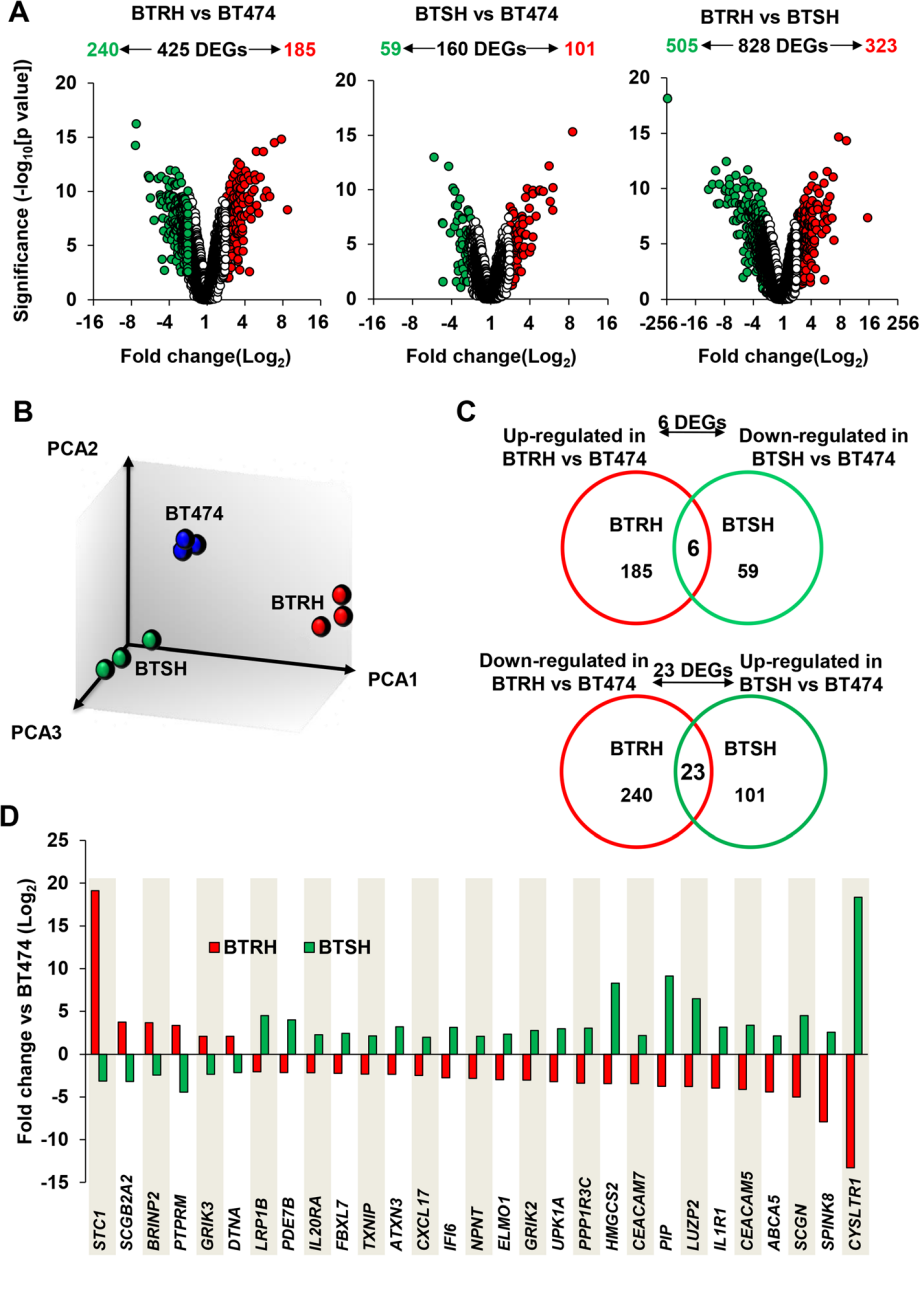
Genomic characterization of trastuzumab-resistant and trastuzumab-hypersensitive cell lines. **A** Volcano plots shows DEGs in BTRH and BTSH versus the parental cell line BT474 and DEGs between BTRH and BTSH cells identified by transcriptomic analysis. Genes are considered differentially expressed if fold change ≥2 and *p*-value ≤0.05. DEGs are represented in green, if downregulated or red, if upregulated; genes in white are out of the cutt-off. **B** Principal component analysis of variance between signal data. Triplicates of each cell line are represented in the same color. **C** Venn diagrams show the number of “inverse amount” DEGs overlapped between BTRH and BTSH versus the parental BT474. Genes upregulated in BTRH versus BT474 were compared to genes downregulated in BTSH versus BT474 and vice versa. **D** The bar chart shows fold change values of 29 “inverse amount” genes in BTRH and BT474 versus the parental cell line BT474

Microarray analyses were performed to explore gene expression differences among the three cell lines. Volcano plot representations showed transcriptomic differences between BT474, BTRH and BTSH cells (Fig. 3A). These analyses indicated that 425 genes were differentially expressed when BT474 and BTRH cells were compared. Of them, 240 were downregulated and 185 genes were upregulated in BTRH. When comparing BT474 to BTSH cells, 160 differentially expressed genes (DEGs) were found. Of them, 59 were downregulated in BTSH while 101 were upregulated. Moreover, a higher number (828) of DEGs were found when BTRH and BTSH were compared (Fig. 3A). Principal component analysis (PCA) of variance from array data indicated different gene expression patterns among cells and clustering of replicates from each cell line (Fig. 3B).

**Fig. 4:**
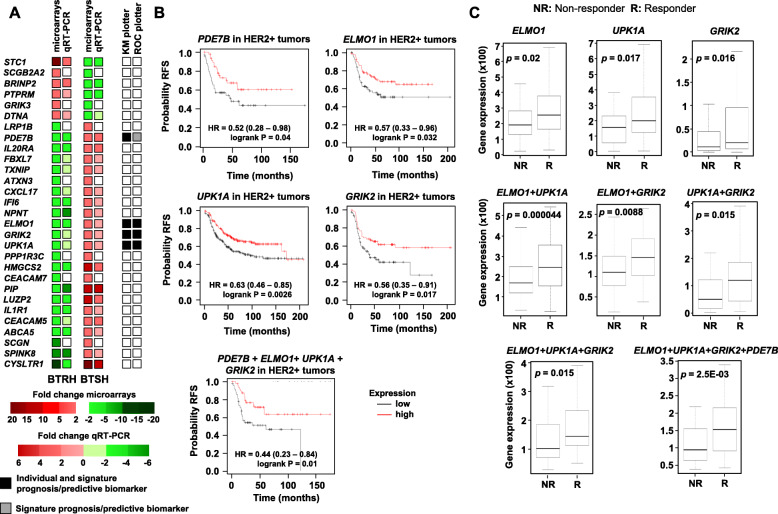
Genes upregulated in BTSH indicates favourable prognosis and trastuzumab response in HER2+ patients. **A** Representation of differential gene expression values from microarrays, qRT-PCR validation and the role as prognosis and predictive biomarkers of “inverse amount” genes found in BTRH and BTSH versus parental BT474 cell line. White squares mean no validation. **B** Kaplan-Meier analysis of the expression of the indicated genes and its correlation with relapse free survival in HER2+ breast cancer patients using the KM plotter bioinformatic tool. The chart below was performed using the multigene classifier tool by selecting the combined gene expression of the four indicated genes. The cut-off value to split patients into low or high expression were lower and upper quartiles. RFS: relapse free survival. **C** Box-plots of genes validated for trastuzumab response in HER2+ breast cancer patients using the pathological complete response database in ROC plotter. Graphs show normalized gene expression in trastuzumab non-responders and responders patients. Gene combinations analysis was assessed by selecting the option signature

We postulated that inversely regulated genes could better identify those genes related to sensitivity/resistance to trastuzumab. Thus, we searched for genes increased in BTRH with respect to BT474 cells, and that also appeared in BTSH but decreased with respect to BT474 cells. On the other hand, we searched for genes downregulated in BTRH but upregulated in BTSH cells. A total of 6 genes were upregulated in BTRH and downregulated in BTSH (Fig. 3C and D). On the other side, 23 genes were downregulated in BTRH and upregulated in BTSH versus BT474 (Fig. 3C and D). qRT-PCR expression studies validated 4 of the 6, and 18 of the 23 genes found deregulated in the expression microarrays (Fig. 4A and Figure S3).

### Impact of genes upregulated in BTSH cells on prognosis and trastuzumab response

The role of the 22 opposing-sign genes on clinical outcome was first evaluated using the online bioinformatic tool KM Plotter [35]. These studies showed that upregulation of genes *PDE7B*, *ELMO1*, *UPK1A* and *GRIK2*, that were upregulated in BTSH and downregulated in BTRH cells, correlated with a favorable prognosis in HER2+ breast cancer patients (Fig. 4A and B). Their combined analysis also showed prognostic relevance. In contrast, none of genes that were upregulated in BTRH and downregulated in BTSH was able to predict patient outcome (Fig. 4A).

The capacity of the genes validated by qRT-PCR to predict response to trastuzumab was then evaluated in a cohort of HER2+ patients treated with trastuzumab. To that end, we used the online tool ROC Plotter [36]. That tool was designed to identify gene expression-based predictive biomarkers using transcriptomic data of a large set of breast cancer patients. The expression of *ELMO1*, *UPK1A* and *GRIK2*, that were upregulated in BTSH and downregulated in BTRH cells, was higher in HER2+ patients who responded to trastuzumab (Fig. 4C). To explore if these genes worked as a predictive trastuzumab response signature, we analyzed their discriminatory potential in previously published clinical samples (Fig. 4C). In particular, ELMO1 (sensitivity = 0.56, specificity = 0.61, AUC = 0.597, *p* = 0.02), UPK1A (sensitivity = 0.66, specificity = 0.55, AUC = 0.615, *p* = 0.017), and GRIK2 (sensitivity = 0.54, specificity = 0.63, AUC = 0.613, *p* = 0.016) reached significance as individual genes. When combining two genes into a signature, all three pairwise comparisons of these were significant and had better AUC values (ELMO1 + UPK1A: sensitivity = 0.67, specificity = 0.37, AUC = 0.659, *p* = 0.000044; ELMO1 + GRIK2: sensitivity = 0.64, specificity = 0.4, AUC = 0.625, *p* = 0.0088; and GRIK2 + UPK1A: sensitivity = 0.56, specificity = 0.31, AUC = 0.615, *p* = 0.015). Finally, the combination of all three genes was also significant (sensitivity = 0.64, specificity = 0.33, AUC = 0.653, *p* = 0.015), a significance of which could be improved by adding PDE7B to the investigated genes (sensitivity = 0.62, specificity = 0.31, AUC = 0.648, *p* = 2.5E-03).

### A trastuzumab-hypersensitive gene signature enriched in patients who responded to dual HER2 blockade

After having performed the above described analyses using the online tools KM Plotter and ROC plotter, to further explore the capacity to predict response to trastuzumab based on the gene expression data obtained from the transcriptomic studies, an additional third analysis was carried out using genomic data available from the PAMELA study [37]. In that study, gene expression data from patients using the nCounter platform was obtained from breast tumors at baseline (before treatment). Patients were treated with dual HER2 blockade (trastuzumab plus lapatinib). PAM50 was evaluated at baseline and day 14 in surgical specimens and pathological response was assessed at surgery. The switch from HER2-enriched to a normal-like subtype at day 14 indicated early tumor response.

**Fig. 5:**
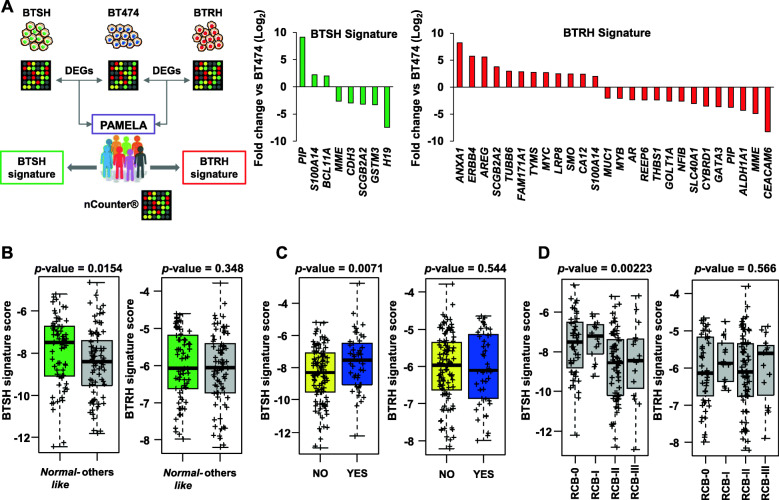
BTSH gene signature is enriched in patients who responded to dual HER2 blockade. **A** A trastuzumab-hypersensitive and a trastuzumab-resistance gene signatures were generated by the overlapping of DEGs identified on microarray analysis performed in BT474, BTRH and BTSH cells with gene expression data from PAMELA’s patients obtained by nCounter platform. Graphs show genes involved in BTSH (left) and BTRH (right) signatures and their fold change compared to parental BT474 cell line. **B**, **C** and **D** Both BTSH and BTRH gene expression signatures were evaluated in PAMELA’s samples as the sum of the mean of the genes from the upregulated list and the mean of downregulated gene list. Correlation of gene signature expression and response to dual HER2 blockade was analyzed by PAM50 at day 14 (**B**), pathological complete response at surgery (**C**) and residual cancer burden score at surgery (**D**). The data is represented as mean of the score of BTSH or BTRH gene signature expression. Each sample is indicated by +. RCB: residual cancer burden

Genes differentially expressed from the transcriptomic studies performed in BT474, BTRH and BTSH cells (Fig. 3A) and also present in the nCounter gene expression array were used to create a BTRH signature (*n* = 26 genes) and a BTSH signature (*n* = 8 genes) (Fig. 5A). We found that the BTSH signature was enriched in patients who responded to dual HER2 blockade, assessed as being normal-like at day 14 (Fig. 5B), pathological complete response at surgery (Fig. 5C) and residual cancer burden (RCB) score of 0-I at surgery (Fig. 5D). However, the trastuzumab-resistant signature was not enriched in patients who did not respond to trastuzumab plus lapatinib.

**Fig. 6:**
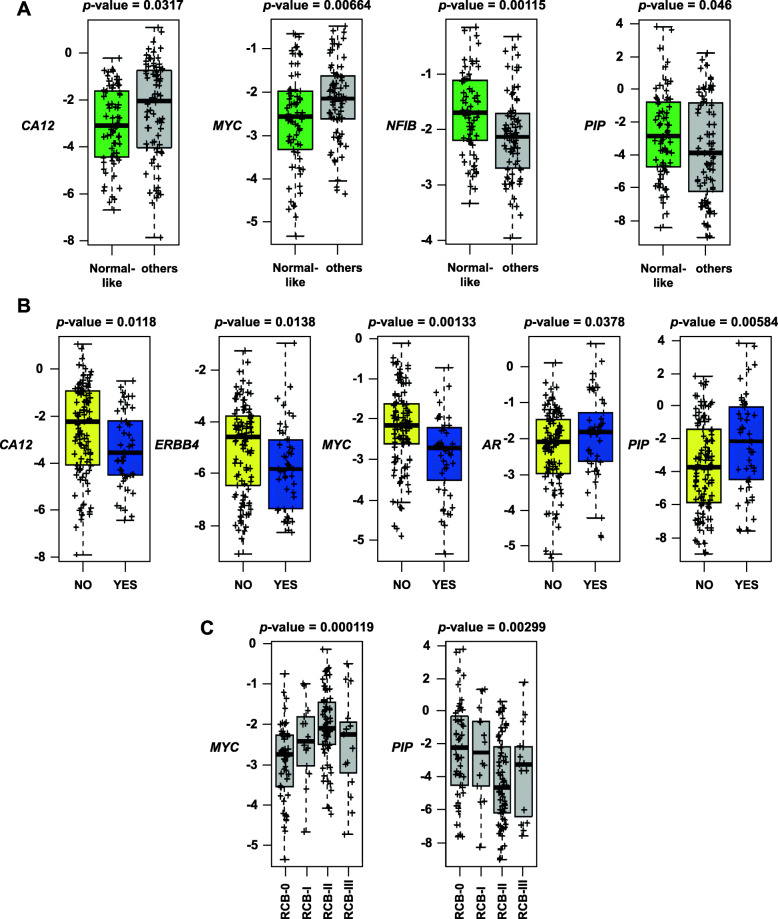
Genes from the BTRH signature predicts trastuzumab resistance in HER2+ patients. The role of individual genes from BTSH and BTRH signatures predicting trastuzumab and lapatinib response was evaluated. Correlation of gene expression and dual HER2 blockade response was assessed by PAM50 at day 14 (**A**), pathological complete response at surgery (**B**) and residual cancer burden score at surgery (**C**). Box plots show expression of indicated genes in each patient sample. Data is represented as the mean of normalized gene expression. Each sample is indicated by +. RCB: residual cancer burden

In addition, the association between the individual genes present in the resistant or sensitive gene signatures and response to trastuzumab and lapatinib was evaluated. *CA12* and *MYC* were upregulated in BTRH and were enriched in patients who did not respond to the treatment (Fig. 6A), *NFIB* and *PIP* were downregulated in BTRH and their expression was upregulated in patients who responded to dual HER2 blockade (Fig. 6A). *PIP* was also upregulated in BTSH cells when compared to the parental cell line and was enriched in patients who responded (Fig. 6A). Genes that worked as predictors of pathological complete response at surgery such as *CA12*, *ERBB4* and *MYC*, all three overexpressed in BTRH, were enriched in patients who did not respond to the treatment (Fig. 6B). *AR* and *PIP* which were downregulated in trastuzumab-resistant cells, indicated pathological complete response in patients (Fig. 6B). Finally, *MYC* expression was increased in patients who exhibited residual cancer burden score of I, II and III (Fig. 6C). *PIP* expression was enriched in patients whose RCB at surgery was 0 or I (Fig. 6C). These results identified some genes from the BTRH signature that associated with poor response to dual HER2 blockade.

## Discussion

Trastuzumab-based therapies represent the gold standard for the therapy of HER2+ breast tumors. However, while most HER2+ tumors respond to these therapies, their response is variable. That circumstance imposes an important clinical question with respect to the expected response to those therapies. Therefore, identification of response markers is of exceptional relevance, particularly considering the follow-up strategy to be carried out. In attempting to progress in that direction, we modelled both resistance and hypersensitivity to trastuzumab to gain insights into determinants that modulate the antitumoral effectiveness of trastuzumab. These studies allowed the identification of individual genes and gene sets that can be used to predict trastuzumab response in patients.

Using different experimental approaches, we were able to isolate HER2+ breast cancer cells with different degrees of sensitivity to trastuzumab. In all the cell line models analyzed, the levels of total or cell surface HER2 and their tyrosine-phosphorylated forms were indistinguishable. That fact excludes that changes in HER2 are responsible for their different sensitivities to the action of trastuzumab. This is relevant since one of the mechanisms proposed to mediate trastuzumab resistance is the expression of a truncated form of HER2 [40–43].

A number of HER2+ breast cancer patients do not benefit from multiple anti-HER2 therapies. That fact could be due to the existence of cross-resistance, leading to tumor progression [44–47]. In line with this, a study published by us demonstrated the existence of cross-resistance between trastuzumab and the TKIs lapatinib and neratinib. BTRH cells were not only resistant to trastuzumab but also they were partially resistant to both TKIs [29]. In contrast, the present study demonstrates that lapatinib and neratinib affected BT474 and BTSH cells survival similarly. We also demonstrate that BTSH cells were hypersensitive to T-DM1 treatment. This may be due to the fact that trastuzumab is part of the backbone of this ADC [48]. In the case of pertuzumab, we did not observe a significant action of the drug on any of the cellular models used. This is particularly noteworthy, since pertuzumab is part of first line treatment in combination to trastuzumab with/without chemotherapy.

To gain insights into the mechanisms responsible for their differential response to trastuzumab, cell cycle and apoptosis experiments were performed. Trastuzumab caused cell cycle arrest at G1 in BT474 and BTSH. Consequently, we found a reduction in the number of cells in S phase in concordance with previous studies [24–26, 49]. In contrast, this effect was not observed in the case of the resistant cell line. In addition, biochemical studies showed p27 upregulation upon trastuzumab treatment in the parental cell line BT474 and the hypersensitive one. Increased levels of that protein in cells treated with trastuzumab has been postulated to mediate the inhibitory properties of the drug on cell cycle progression [49–51]. A clearly distinctive feature between parental and BTSH cells with respect to the response to trastuzumab was the sensitivity of the latter cell line to cell death induced by the drug. While markers of apoptosis indicated that this is the route by which trastuzumab acts on hypersensitive cells, the precise mechanism by which trastuzumab promotes apoptotic cell death remains elusive.

**Fig. 7:**
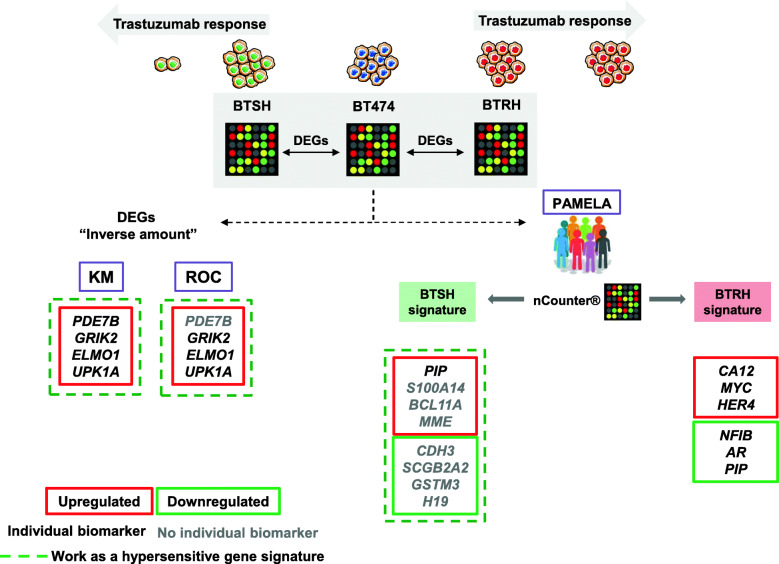
Biomarkers of clinical outcome and trastuzumab responsiveness. DEGs in BTSH and BTSH compared to parental BT474 cell line were determined by microarrays analyses. To identify biomarkers of clinical outcome and trastuzumab response to two different strategies were followed. First, the role of DEGs with “inverse amount” on prognosis and trastuzumab response in HER2+ breast cancer patients was evaluated by KM plotter and ROC plotter, respectively. On the other hand, DEGs were overlapped with transcriptomic data from PAMELA clinical trial patients’ tumor samples and response to dual HER2 blockade was assessed

Once the different cellular models of sensitivity to trastuzumab were characterized, transcriptomic data was generated in attempting to predict trastuzumab response. We selected significant genes inversely regulated in BTRH and BTSH with respect to the parental cell line BT474. These identified genes were analyzed for their prognostic impact using the KM Plotter tool [35]. These studies showed that 4 genes (*PDE7B, ELMO1, UPK1A* and *GRIK2*) out of 22 validated genes, which were upregulated in BTSH, indicated good prognosis in HER2 positive breast cancer patients, either individually or working as a gene expression signature (Fig. 7). In the search for predictive biomarkers of trastuzumab response we used the ROC plotter tool [36]. These studies allowed the identification of 3 genes (*ELMO1, UPK1A* and *GRIK2*), all of them highly expressed in BTSH cells, which were upregulated in patients who responded to trastuzumab. Together, the above studies allowed the identification of several gene sets and individual ones with potential clinical utility as biomarkers of trastuzumab response and good prognosis in HER2+ breast cancer.

With the aim of facilitating translation of our findings into the clinical setting, cell line expression data from microarrays was compared to expression data from tumors of a cohort of 151 HER2 positive patients belonging to the PAMELA clinical trial [37](Fig. 7). Deregulated genes obtained from the microarray analyses performed on BTRH and BTSH were analyzed for their presence and amount in the nCounter platform, used to obtain gene expression data in the patients of that clinical trial. Following this approach, BTRH and BTSH signatures were expressed by patients’ tumors at baseline. We found that the hypersensitive gene expression signature was enriched in patients who responded to the HER2 dual blockade. These results suggested that some genes expressed by the BTSH model could predict good trastuzumab response. Additional studies evaluating single genes present in the BTRH signature, indicated that some of these genes were able to predict response to trastuzumab. On the one hand, *CA12* and *MYC* were upregulated in patients who did not respond. In terms of pathological complete response *CA12*, *ERBB4* and *MYC* were enriched in patients who did not achieve that response. Moreover, *AR* and *PIP* were downregulated in patients who did not exhibit pCR at surgery in concordance with its lesser expression in trastuzumab-resistant cells. Taking all these findings into account, the results suggested that *CA12, MYC, ERBB4, NFIB, PIP* and *AR*, genes present in the BTRH signature could potentially predict trastuzumab plus lapatinib response in HER2 positive patients. It is important to outstand that *PIP* was also involved in the hypersensitive signature, being upregulated in BTSH cells and HER2 positive patients who responded to dual HER2 blockade.

## Conclusions

In conclusion, our work shows the predictive value of a selected set of genes on both clinical outcome and trastuzumab responsiveness in patients with HER2+ breast cancer. These results open the possibility of further verifying the predictive value of those genomic markers in larger and well controlled clinical trials. Moreover, the possibility of detecting the genes/signatures in liquid biopsy specimens should also be explored.

## Supplementary Information

**Additional file 1: Figure S1.** Susceptibility of HER2+ breast cancer cell lines to trastuzumab. **A)** Effect of trastuzumab on SKBR3 cells proliferation. Cells were treated with trastuzumab for 7 days and cell number was measured by cell counting experiments. Data is represented as mean ± SD. **B)** and **C)** Evaluation of trastuzumab response in the 54 clones derived from the parental BT474 cell line. Cells were treated with trastuzumab for 7 days and cell number was determined by cell counting assays. Data is represented as mean ± SD, normalized to untreated controls of each clone. Blue squares indicate the response to trastuzumab in BT474 cells. **B)** Above 70% of proliferation, clones were considered resistant (red arrow) and below 30% sensitive (green arrow) to trastuzumab, respectively. **D)** Trastuzumab decreases ST35 cells compared to BT474. The graph shows BT474 and ST35 proliferation in the presence of trastuzumab for 7 days. Data is represented as mean ± SD, normalized to untreated controls.

**Additional file 2: Figure S2.** Effect of trastuzumab treatment on cell cycle progression and apoptosis. **A)** Effect of trastuzumab on cell cycle in BT474, BTRH and BTSH was evaluated by flow cytometry after 6 days trastuzumab treatment propidium iodide staining. Percentage of cells in G0/G1 (red) and S plus G2/M (blue) phases of one representative experiment was indicated. Trastuzumab causes a slightly increase in dead cells in BT474 **(B)** and SKBR3 **(C)** cells. Cells were treated with trastuzumab for 96 h and double stained with annexin V-FITC and propidium iodide. The percentage of cells in each quadrant is indicated. Cell viability was analyzed by flow cytometry. The percentage of viable and non-viable cells was represented as the mean ± SD of two independent experiments. ANN: Annexin V-FITC; C: control; PI: propidium iodide; T: trastuzumab.

**Additional file 3: Figure S3.** Inverse amount gene expression validation by qRT-PCR. Detection of mRNA levels of the indicated genes in BTRH and BTSH. Gene levels were normalized to GAPDH and relativized to those from parental BT474 cell line. The graph represents the mean ± SD of data from three independent experiments.

## Data Availability

The dataset supporting the conclusions of this article is available in GEO repository (GSE184655).

## Abbreviations

AUC: Area under the curve
DEGs: Differentially expressed genes
HER2+: HER2 positive
KM plotter: Kaplan Meier Plotter
PCA: Principal component analyses
pCR: Pathological complete response
PI: Propidium iodide
T-DM1: Trastuzumab-emtansine
TKIs: Tyrosine kinase inhibitors
