# Supplementary figures and images for: Modelling hypersensitivity to trastuzumab defines biomarkers of response in HER2 positive breast cancer
Source: J Exp Clin Cancer Res. 2021 Oct 7;40:313. doi: 10.1186/s13046-021-02098-z (PMC8496101; doi:10.1186/s13046-021-02098-z)

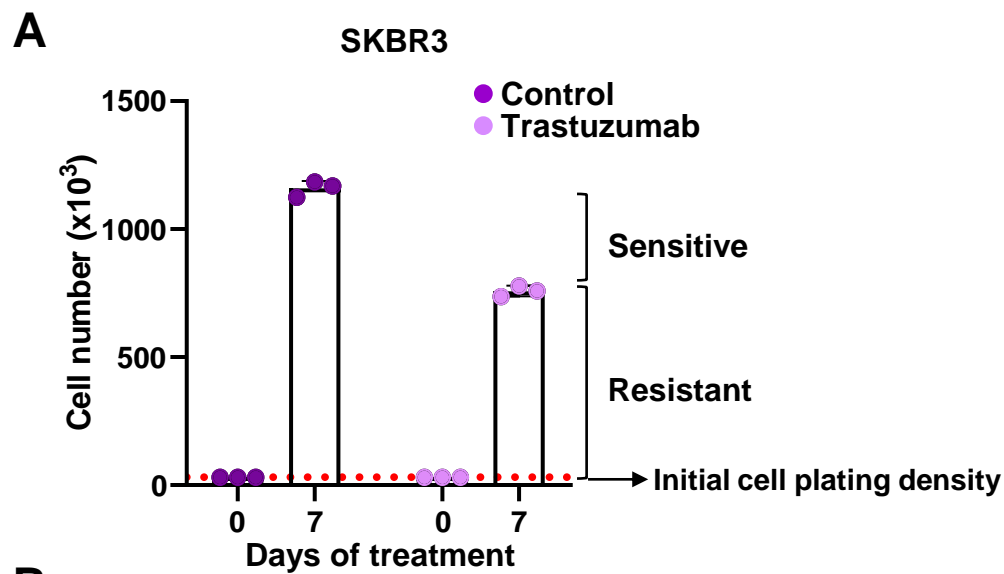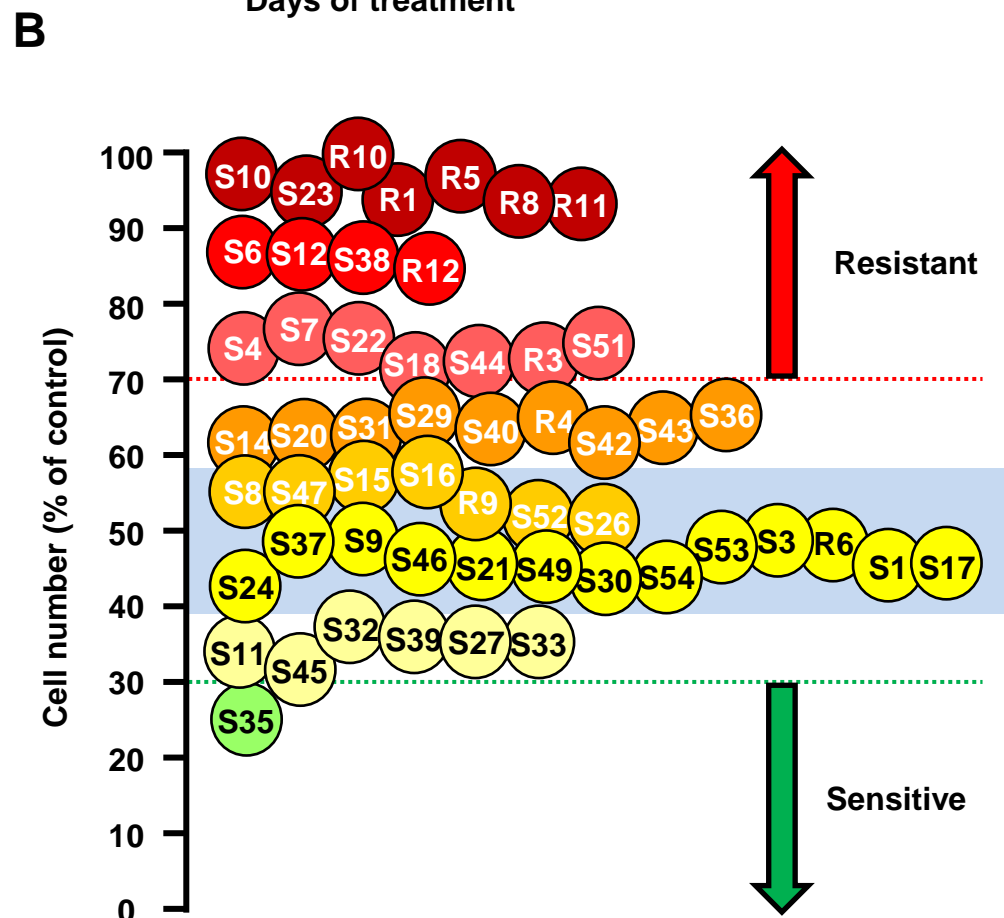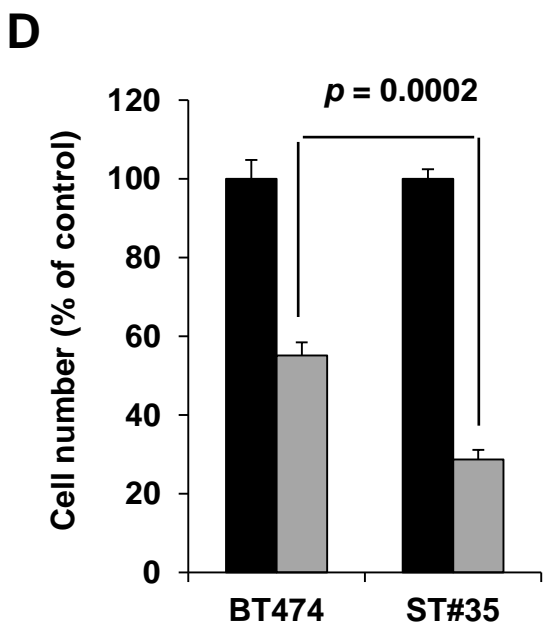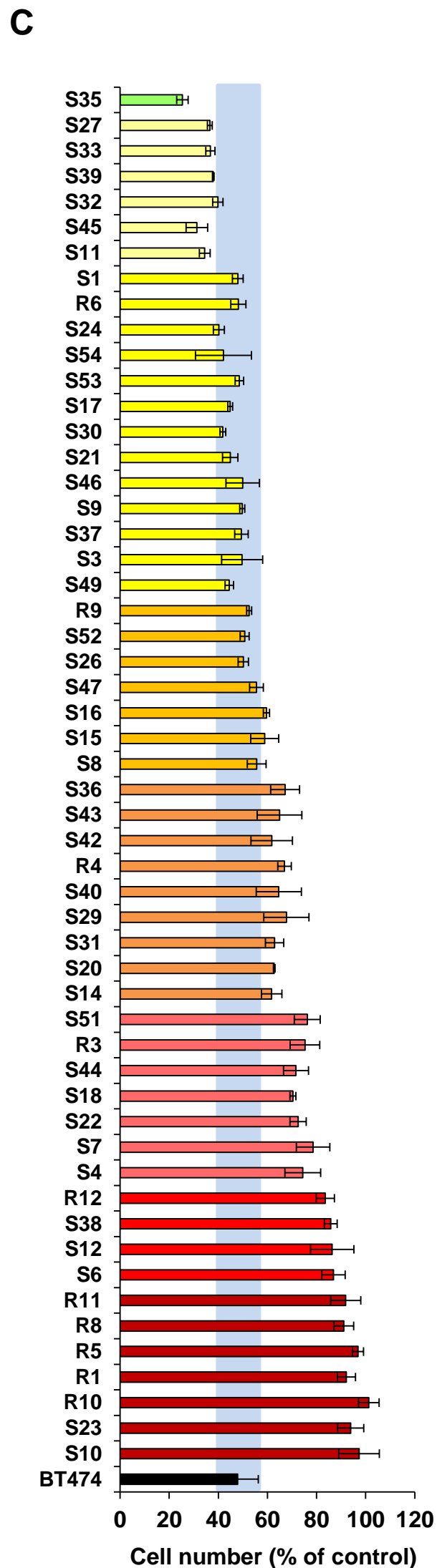

Figure S1

Supplement: Supplementary file 1 — Additional file 1: Figure S1. Susceptibility of HER2+ breast cancer cell lines to trastuzumab. A) Effect of trastuzumab on SKBR3 cells proliferation. Cells were treated with trastuzumab for 7 days and cell number was measured by cell counting experiments. Data is represented as mean ± SD. B) and C) Evaluation of trastuzumab response in the 54 clones derived from the parental BT474 cell line. Cells were treated with trastuzumab for 7 days and cell number was determined by cell counting assays. Data is represented as mean ± SD, normalized to untreated controls of each clone. Blue squares indicate the response to trastuzumab in BT474 cells. B) Above 70% of proliferation, clones were considered resistant (red arrow) and below 30% sensitive (green arrow) to trastuzumab, respectively. D) Trastuzumab decreases ST35 cells compared to BT474. The graph shows BT474 and ST35 proliferation in the presence of trastuzumab for 7 days. Data is represented as mean ± SD, normalized to untreated controls. [file 13046_2021_2098_MOESM1_ESM.pdf]

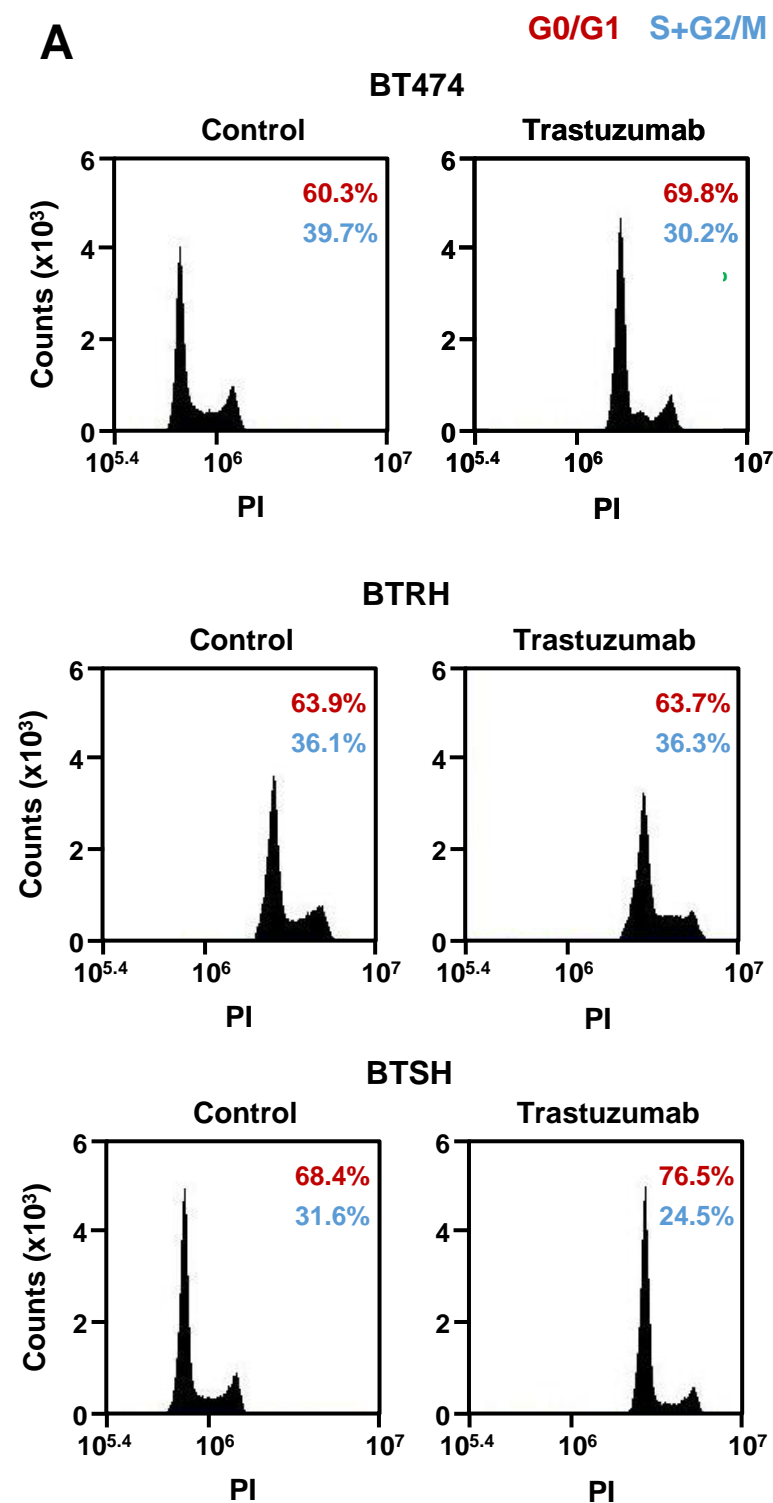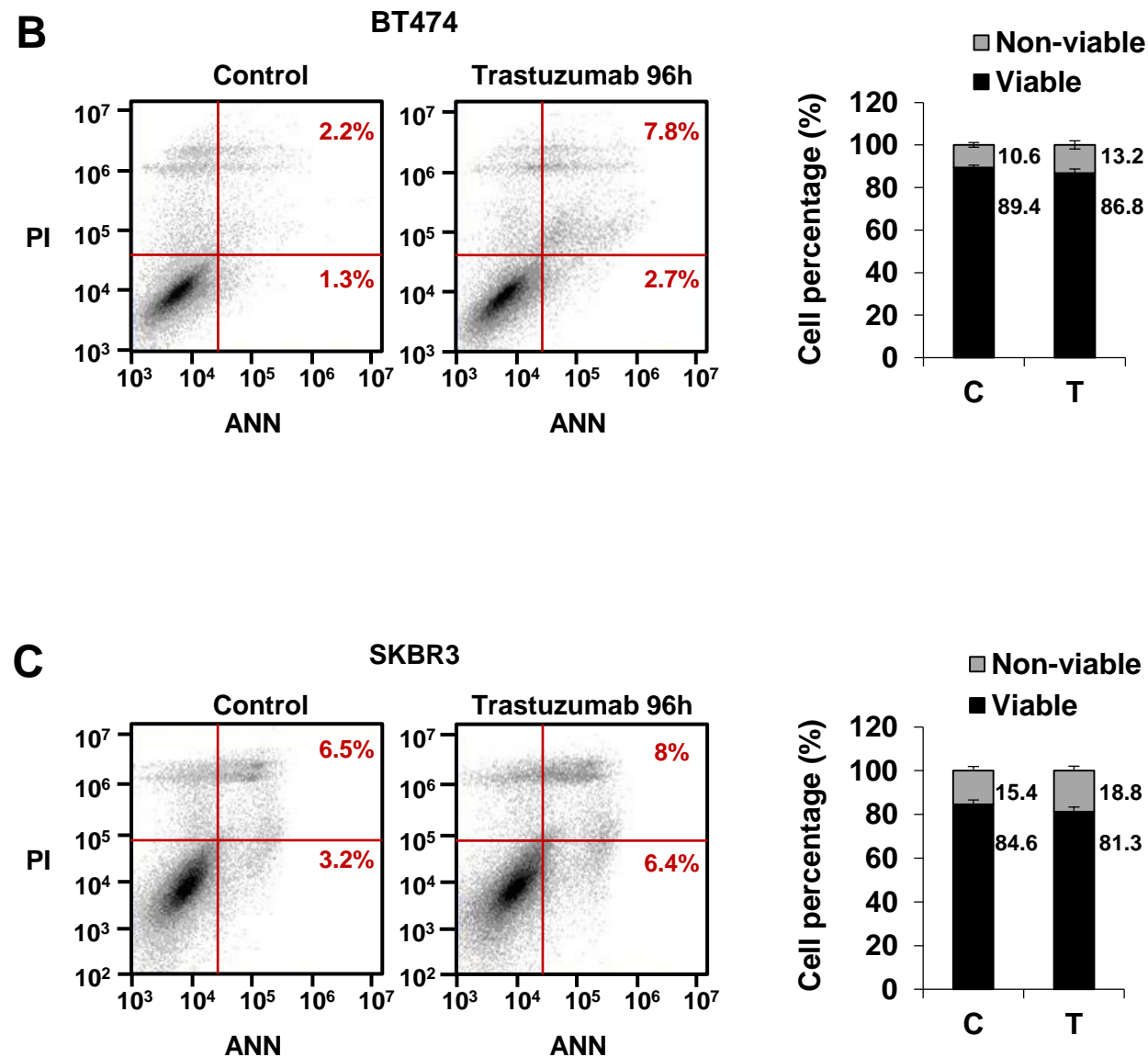

Figure S2

Supplement: Supplementary file 2 — Additional file 2: Figure S2. Effect of trastuzumab treatment on cell cycle progression and apoptosis. A) Effect of trastuzumab on cell cycle in BT474, BTRH and BTSH was evaluated by flow cytometry after 6 days trastuzumab treatment propidium iodide staining. Percentage of cells in G0/G1 (red) and S plus G2/M (blue) phases of one representative experiment was indicated. Trastuzumab causes a slightly increase in dead cells in BT474 (B) and SKBR3 (C) cells. Cells were treated with trastuzumab for 96 h and double stained with annexin V-FITC and propidium iodide. The percentage of cells in each quadrant is indicated. Cell viability was analyzed by flow cytometry. The percentage of viable and non-viable cells was represented as the mean ± SD of two independent experiments. ANN: Annexin V-FITC; C: control; PI: propidium iodide; T: trastuzumab. [file 13046_2021_2098_MOESM2_ESM.pdf]

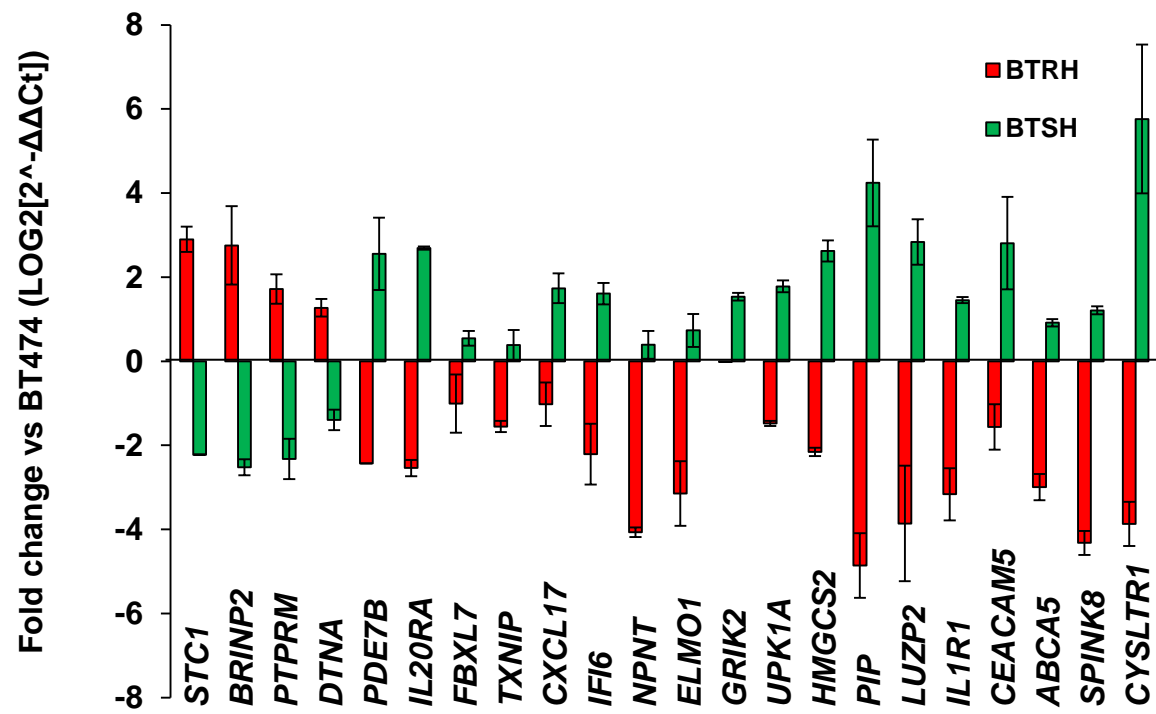

Figure S3

Supplement: Supplementary file 3 — Additional file 3: Figure S3. Inverse amount gene expression validation by qRT-PCR. Detection of mRNA levels of the indicated genes in BTRH and BTSH. Gene levels were normalized to GAPDH and relativized to those from parental BT474 cell line. The graph represents the mean ± SD of data from three independent experiments. [file 13046_2021_2098_MOESM3_ESM.pdf]
